# Microarray Dot Electrodes Utilizing Dielectrophoresis for Cell Characterization

**DOI:** 10.3390/s130709029

**Published:** 2013-07-12

**Authors:** Bashar Yafouz, Nahrizul Adib Kadri, Fatimah Ibrahim

**Affiliations:** Medical Informatics and Biological Micro-Electro-Mechanical Systems (MIMEMS) Specialized Laboratory, Department of Biomedical Engineering, Faculty of Engineering, University of Malaya, 50603 Kuala Lumpur, Malaysia; E-Mails: bashar.yafouz@siswa.um.edu.my (B.Y.); fatimah@um.edu.my (F.I.)

**Keywords:** dielectrophoresis, Lab-on-a-Chip, dot electrode, BioMEMS, particle manipulation

## Abstract

During the last three decades; dielectrophoresis (DEP) has become a vital tool for cell manipulation and characterization due to its non-invasiveness. It is very useful in the trend towards point-of-care systems. Currently, most efforts are focused on using DEP in biomedical applications, such as the spatial manipulation of cells, the selective separation or enrichment of target cells, high-throughput molecular screening, biosensors and immunoassays. A significant amount of research on DEP has produced a wide range of microelectrode configurations. In this paper; we describe the microarray dot electrode, a promising electrode geometry to characterize and manipulate cells via DEP. The advantages offered by this type of microelectrode are also reviewed. The protocol for fabricating planar microelectrodes using photolithography is documented to demonstrate the fast and cost-effective fabrication process. Additionally; different state-of-the-art Lab-on-a-Chip (LOC) devices that have been proposed for DEP applications in the literature are reviewed. We also present our recently designed LOC device, which uses an improved microarray dot electrode configuration to address the challenges facing other devices. This type of LOC system has the capability to boost the implementation of DEP technology in practical settings such as clinical cell sorting, infection diagnosis, and enrichment of particle populations for drug development.

## 1. Introduction

The trend toward point-of-care (POC) systems has grown dramatically in the past two decades. New diagnostic tools are needed to meet the increasing demand for fast, reliable and cost-effective diagnostic devices. These new tools would replace currently available tests that can only be conducted in fully equipped diagnostics laboratories [1]. Many research groups have proposed models for POC systems that are implemented in microfluidic-based platforms, based on a wide range of available technologies. Miniaturized laboratory equipment has been designed to achieve better reaction efficiency and faster results, while being more portable and consuming fewer reagents.

One of the platforms used in microfluidic devices is the lab-on-a-chip (LOC), which has great potential for use in automated bio-microfluidic diagnostic systems. Various diagnostic techniques have been employed to effectively integrate multiple microfluidic components into fully automated LOC systems that can perform sophisticated biomedical analyses [2]. Specifically, capillary driven microfluidics, multilayer soft lithography, multiphase microfluidics, electrowetting-on-dielectric mechanisms, electrokinetics, and centrifugal microfluidics are the platforms with the most potential for incorporating microfluidics into a variety of biomedical engineering applications [3]. Each approach has unique advantages and disadvantages. This paper discusses dielectrophoresis (DEP) (specifically electrokinetics), which offers a number of positive features that many other available techniques are unable to provide.

In this paper, we demonstrate the potential of the microarray dot electrode geometry to generate the DEP effect for cellular characterization and manipulation. Furthermore, we review several state-of-the-art LOC devices that have been described in the literature to create DEP systems. Finally, we present our recently designed LOC device, which works with the improved microarray dot electrode configuration to address the challenges faced by other devices.

## 2. Lab-on-a-Chip (LOC)

Various LOC designs have been developed in the past three decades for use in several different applications. The strengths of such systems include a reduced requirement for samples and reagents, fast and high-throughput results, low power consumption, a reduced contamination risk and a high degree of parallelization [1,3]. These designs take advantage of the great developments occurring in microfabrication techniques.

Masuda *et al.* were one of the first groups to use DEP in an LOC device [4]. They designed a “fluid integrated circuit,” which was used to manipulate cells and separate them into different outlets. The proposed tool enabled automated single cell manipulation and device miniaturization by combining multiple cell-handling components, such as micropumps and cell-fusion electrodes, onto one substrate.

Gascoyne *et al.* described a device for separating certain cancerous cells from blood in a dielectric affinity column [5]. The device involved two parallel glass walls, in which the lower portion of the glass contained an interdigitated electrode. Three holes were drilled in the top chamber wall to play the roles of inlet and outlet ports for cell suspensions and elution buffers. A gasket chamber of a 100 μm thickness was manufactured using Teflon. This technique, when integrated with other diagnostic or cell separation applications, increased the overall efficiency and sensitivity of the device.

MDA-435 cells, which are human breast cancer cells, and peripheral blood mononuclear cells were separated according to their dielectric properties by a device developed by Gasperis *et al.*[6]. That device combined 2D dielectrophoretic forces with field flow fractionation to manipulate cells efficiently. The device consisted of a 250-μm thick latex gasket sandwiched between top and bottom plastic plates. The electrical connections to the electrodes were linked via pressure-loaded metal wires. This device could be integrated with other microfluidic elements, such as a micro polymerase chain reaction (micro-PCR) system and capillary electrophoresis systems, to create a preliminary stage for sample preparation.

Li *et al.* [7] proposed a highly accurate device to manipulate *Listeria innocua* bacteria with DEP. The DEP effect took place in a rectangular electrode chamber, which was built by attaching silicone rubber to a glass substrate equipped with interdigitated microelectrodes. Such accurate yet simple LOC arrangements have great potential to be implemented in diagnostic applications.

A device developed by Fatoyinbo *et al.* rapidly determined the dielectric properties of biological cells [8]. The device consisted of a 4 × 4 dot-patterned gold microarray with a parallel ground ITO microelectrode on top. The microarray was designed so that each dot could be energized separately. The gasket chamber was fabricated using a UV-curing photopolymer resin. Overall, their design measured cell electrophysiology at a nearly real-time speed.

The above reviewed works are capable of successfully manipulating and separating their intended cell populations; however, most of them are contained by laboratories and have yet to gain worldwide recognition by the biotechnology industry. Their limitations are due to fabrication constraints and difficulties, primarily cost factors.

## 3. Dielectrophoresis

DEP was chosen among other various approaches to manipulate cells due to several advantages. Features of DEP include non-invasiveness, high selectivity and efficacy on small scales, label-free manipulation, low costs and well-established fabrication techniques [9].

DEP, a phenomenon that can be used to manipulate and characterize polarizable particles, has been shown to work on a wide range of constituents since its discovery by Pohl [10]. It is a novel technique to separate living and dead cells by taking advantage of each bioparticle's unique electrical properties [11]. DEP describes the movement of polarizable particles under non-uniform electric fields.

By applying electrical forces at the micro level, researchers can determine the forces generated on cells, which depend on the electrical phenotype of the cell. This information can be used to determine a cell's electrical properties (conductivity and permittivity) as a function of the applied signal frequency, creating a plot known as a “DEP spectrum,” making DEP a powerful tool for cell characterization studies. Different cells exhibit different crossover frequencies which are the frequency values where a cell's DEP effect changes from positive to negative DEP or *vice versa* [12].

The electrical phenotype of a cell is primarily determined by its total net charge and polarizability. Electrical phenotypes are valuable because they correlate to biological variations in cells. Therefore, DEP can be used to characterize two biologically different cells [13].

One of the core strengths of DEP is that the characterization of different cells depends only on their dielectric properties, which are controlled by the cell's individual phenotype. Hence, the process of DEP characterization does not require specific tags or involve chemical reactions [14].

Since its discovery in the 1950s, DEP has been employed in a wide variety of applications. It has been used to manipulate viruses [15–19], proteins [20–22], bacteria [23–26], DNA [27–29], spores [30,31], algae [32,33], and parasites [34,35]. DEP has also been used in research on apoptosis [36,37] and cell lysis and viability [38–40]. Moreover, the phenomenon has been used to manipulate micro-sized polystyrene particles [41], nano-sized latex particles [42] and biopolymers [43]. DEP has been employed extensively to characterize various mammalian cells such as neurons [44], leukemia cells [45], yeast cells [46], platelets [47], cancer cells [48] and sperm cells [49].

### 3.1. Theory

**Figure 1.**
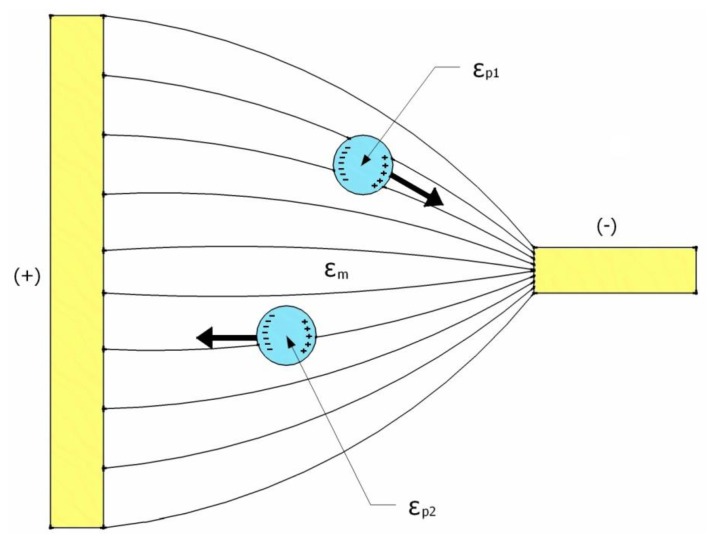
A schematic illustration of the responses of polarizable particles to a non-uniform electric field, provided that ε_p1_ > ε_m_ > ε_p2_; ε_m_, ε_p1_ and ε_p2_ are the permittivities of the medium, particle 1 and particle 2, respectively.

Applying a non-uniform electric field to polarizable particles that are placed in a conductive medium produces a DEP force. The magnitude and direction of the DEP force depends on the relative polarizabilities of the particle and of the surrounding medium [50]. The DEP force acting on a spherical particle can be expressed by the following equation [51]:

(1) 〈F→DEP〉=2πr3ɛoɛmRe[K(ω)]∇E2

where *ε*_o_ is the permittivity of free space, *ε*_m_ is the permittivity of the surrounding medium, r is the particle radius, ∇E is the electric field gradient and Re[K(ω)] is the real part of the Clausius-Mossotti factor. The Clausius-Mossotti factor is defined as follows:

(2) $$K(\omega )=\frac{{ɛ}_{p}^{*}-{ɛ}_{m}^{*}}{{ɛ}_{p}^{*}+2{ɛ}_{m}^{*}}$$

where ε* is the complex permittivity and subscripts p and m denote the particles and the medium, respectively. The complex permittivity ε* is described by the below equation:

(3) $${ɛ}^{*}=ɛ-j\frac{\sigma }{\omega }$$

where ε is the permittivity, $j=\sqrt{-1}$, σ is the conductivity and ω is the angular frequency of the applied AC electric field. The value of Re[K(ω)] for a sphere ranges between −0.5 and 1, and it depends on the frequency of the applied AC electric field and the relative polarizability between the particle and its surrounding medium [51]. Thus, particles can be induced to travel in a specific direction by carefully selecting these parameters. As illustrated in Figure 1, when the permittivity of the particle is higher than that of the surrounding medium, Re[K(ω)] > 0, and the particles have a positive DEP and move up the electric field gradient to the higher region. On the other hand, when the permittivity of the particle is lower than that of the surrounding medium, Re[K(ω)] < 0, and the particles travel down the electric field gradient to the lower region as a result of the negative DEP effect.

## 4. Electrode Geometry

The non-uniform electric field required to develop the DEP effect is generated by either electrode or electrodeless (insulator-based) DEP devices. Electrodeless DEP employs spatially non-uniform insulating constriction to generate a high electric field gradient with a local maximum [52]. The advantage of such electrodeless devices is found in the fact that its structure is mechanically robust and chemically inert [53]. However, these devices require a high voltage supply and the channel length is limited (∼10 mm) [54]. On the other hand, electrode-based DEP devices provide large trapping areas for cell aggregation and do not require high driving voltage; since the electric field is produced by electrodes on the micron scale. Another advantage of miniaturizing the electrodes is the corresponding decrease in the temperature rise caused by joule heating; ∇T ∼ L^2^|E|^2^, where L is the length that characterizes the electric field variations, so a smaller L means a smaller ∇T provided that the applied electric field is fixed [55].

Various electrode geometries have been proposed in the literature, but they can generally be categorized into two main groups: planar and 3D electrodes. Planar electrodes are typically patterned on the bottom of a microchannel using conventional lithography techniques. Examples of planar electrode designs include interdigitated [56], castellated [57], spiral [58], curved [59], oblique [60], quadrupole [61] and matrix [8]. On the other hand, 3D electrodes can be designed on the bottom, the bottom and top, or the sidewalls of a microchannel, but these designs require complicated techniques. Examples of 3D electrode designs include a grid pattern [62], microwells [63], DEP-wells [64], extruded patterns [65], a sidewall pattern [66] and a top-bottom pattern [67].

This variety in electrode geometry has evolved to address different research tasks. The overall purpose of 3D electrode designs is to perform characterization studies on large populations of particles. For example, interdigitated electrodes are very popular for manipulating certain populations of particles depending on their characteristic electrical properties; on the other hand, a grid electrode is designed to precisely control the physical motions of individual particles [14]. Hence, the electrode geometry to be used is determined by the goal of the study.

One of the early non-uniform electric field designs of Herbert Pohl, the pioneer of DEP, was built by inserting a wire into a glass tube, with another wire surrounding the inner wall of the glass tube [68]. However, this device required a high electric potential and could only manipulate particles larger than 1 micron due to the Joule heating effects. Another drawback to this design is that its electrodes only quantify positive DEP, since the target particles were manipulated to move only toward the wires not away of them.

A quadrupole electrode, made of four electrodes facing each other to form an inner defined area, has been a popular electrode configuration choice for particle trapping and DEP manipulation applications [17,69,70]. Recently, Guan *et al.* used a quadrupole electrode to trap charged particles in aqueous solution [71]. This type of electrode has many advantages; however, it increases the applied electric field, resulting in a divergence of the targeted particles so that they travel away from the center of the four electrodes. To avoid this limitation, Voldman *et al.* proposed the extruded quadrupole electrode to manipulate particles using stronger DEP forces [72]. However, these extruded quadrupole electrodes, similar to other 3D electrodes, include complex fabrication processes.

In general, most of these electrode geometries generate complicated and asymmetrical electric fields, making it difficult to measure the DEP force on the particles and extract their dielectric properties. Hence, there was a need to develop an electrode with simpler and more uniform geometry to address these challenges.

## 5. Microarray Dot Electrode

There are many advantages to the dot electrode design, making its configuration unique and more practical than other existing designs:

- *Confined Region of Analysis*: The round structure of the dot electrode creates a well-defined and enclosed region of analysis. This attribute enables the holding and manipulation of individuals and populations of cells in defined locations.
- *Simple Fabrication*: A dot electrode is a planar 2D electrode with a simple fabrication procedure that utilizes the standard lithography technique. This feature gives the dot electrode design a competitive advantage over existing 3D electrode designs, which require complex fabrication steps. The dot gold electrode, for example, is fully fabricated in approximately 3 hours by the photolithography technique (provided that the photomask of the electrode pattern is available).
- *Strong Field*: While other 2D electrode designs suffer from weak trapping forces, micro-array ring-dot electrodes allow for the creation of multiple capture sites, each with strong trapping [73]. Furthermore, previous simulation works were conducted to explore the electric field over the dot electrode using numerical analysis [74,75], and these works have confirmed its effective electric field penetration.
- *Integrity*: The round pattern of the dot electrode can be used for the probe surfaces of antibodies and biosensors in diagnostic devices. Piezoelectric, optical and electrochemical biosensors can be incorporated with DEP technology to better characterize and manipulate cells [76,77]. In addition, because the dot electrode geometry is simple (with the resultant image being circular), no registration is needed between the images and the electric field template [75].
- *Scalability*: The number of dots in the electrode can be scaled up to create larger arrays, allowing more regions of analysis and reducing the time needed to generate the DEP spectrum.
- *Symmetry*: The dot electrode design is geometrically symmetrical. As a result, the electrode can manipulate cells regardless of the flow direction used for their injection.
- *Re-dispersion*: When removing the electric field from the dots, particles were observed to travel back across the dot, ending in a near-homogeneous distribution after a period much shorter than that predicted for diffusion alone [75]. This phenomenon enabled serial experiments to obtain a complete DEP spectrum to be conducted rapidly because there is no need for external intervention to redistribute the particles over the dots after each experiment.

**Figure 2.**
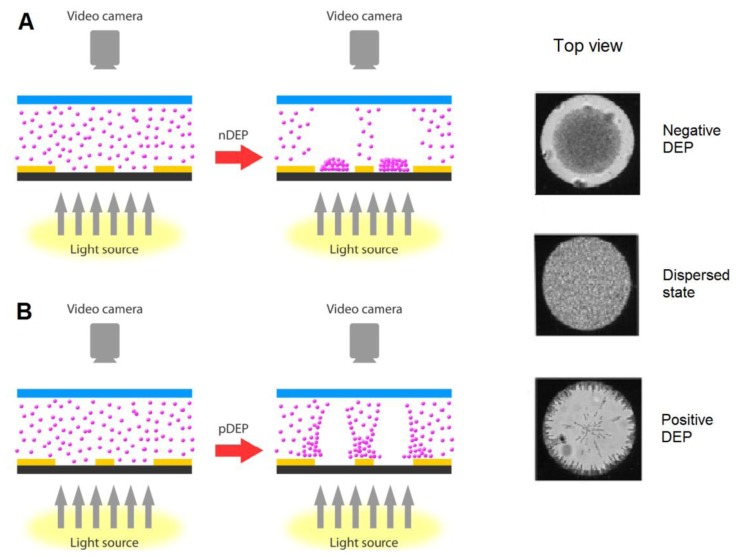
A schematic diagram of the movement of particles within the dot microelectrode device when experiencing (**A**) negative DEP, (**B**) positive DEP. Reproduced with permission from [75].

Figure 2 illustrates a schematic diagram for the DEP effect caused by the dot electrode. When supplying the electrodes with low frequency signal (∼10 kHz), particles exhibit n-DEP and accumulate at the center of the dot aperture. On the other hand, p-DEP occurs when applying high frequency signal (∼1 MHz) causing the particles to be cleared from the center of the dot aperture and attracted to the electrode edge.

A circular “ring-dot” electrode geometry was proposed by Taff and Voldman for bioparticle trapping and sorting using p-DEP [73]. The ring-dot electrode geometry incorporates an outer ring electrode and an inner dot electrode that lies on a different metal layer. This electrode design maintains a strong and spatially localized electric field.

Fatoyinbo *et al.* developed a dot microsystem to rapidly determine the dielectric properties of biological cells [75]. Their device consisted of a dot-patterned gold microarray with parallel ground microelectrode on top, made of indium tin oxide (ITO) material. They linked the electrical properties of the particles to the shifts in light transmission through the dots and computed their results from an analysis of digital images. They successfully obtained the DEP spectrum of 26 data points in approximately 15 minutes.

Recently, Fatoyinbo *et al.* successfully recorded DEP events very close to real-time using independently addressable dot microelectrodes in an array format [8]. These simultaneously energized dots had different frequencies and increased the rate of data acquisition, bringing the field a step closer towards obtaining a synchronous DEP spectrum.

Fatoyinbo *et al.* also reported a re-dispersion effect on the particles when the electric field was switched off [75]. They noted that this effect was significantly easier to observe when the size of the dot decreased and the particles stock concentration increased. As a result, almost real-time electrophysiology has been conducted successfully by taking advantage of this phenomenon [8]. This feature makes the dot electrode design a potential choice to develop a rapid and automated LOC device for biomedical applications. Dot microelectrode geometry possesses special features that can enhance the exploitation of the DEP phenomenon.

## 6. Electrode Fabrication

The uniqueness of dot electrodes is in their planar geometry nature. Its flat geometry gives the dot electrode a crucial advantage over 3D electrodes because it is easier to fabricate. There are number of techniques exist for the fabrication of microfluidic devices, including wet etching, reactive ion etching, conventional machining, soft lithography, hot embossing, injection molding, laser ablation, *in situ* construction, and plasma etching [78]. However, photolithography is considered the basis for most of these processes. Two-dimensional microelectrodes such as the dot microelectrode can be fabricated using the simple photolithography technique. The overall photolithography process requires only 3 hours to fabricate planar electrodes, assuming the photomask which carries the electrode geometry is available.

The electrode geometry is designed first, using any computer-aided design (CAD) software that sustains rigorous dimensions and shapes at a very high resolution. The pattern of the electrode is printed onto soda lime glass, quartz or polyester film to make the photomask; the critical dimension of the electrode geometry determines the photomask material. While quartz and soda lime glass maintain high quality photomasks that have high stability, emulsion polyester film provides cheap photomasks that can preserve features up to a few microns, depending on the resolution of the mask writer.

Table 1 summarizes the protocols used in fabricating a gold-coated glass microelectrode using the photolithography technique. The geometry pattern is transferred from the photomask to the substrate utilizing a photoresist material. There are two types of photoresist: positive and negative. Table 1 describes the photolithography process using a positive photoresist; they have become popular because they offer better process control for small geometry features. The gold etchant solution referenced in Table 1 was prepared by mixing KI, I_2_ and H_2_O in a ratio of 4 g:1 g:40 mL.

**Figure 3.**
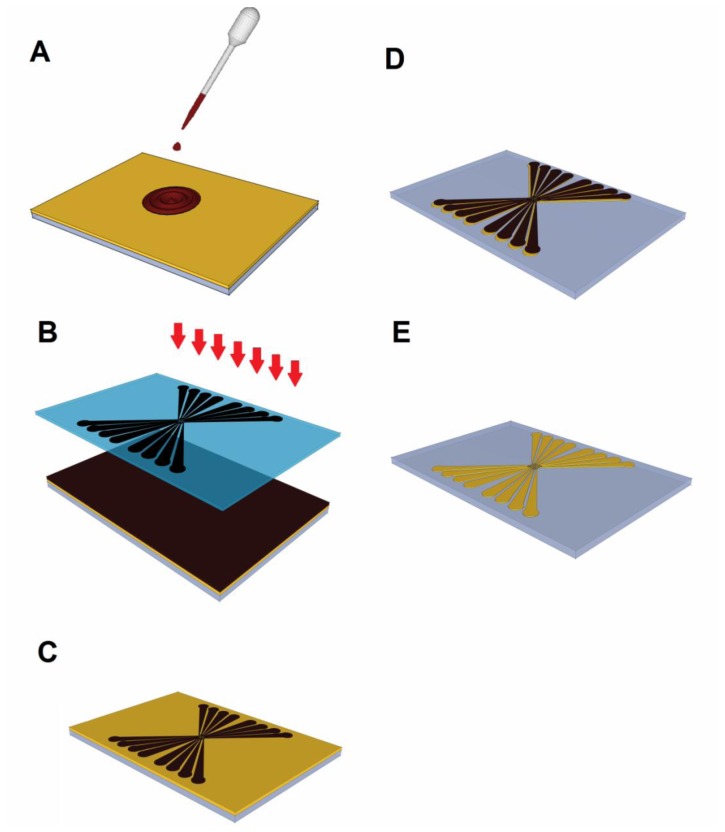
Images of the key steps in the fabrication of planar electrodes by photolithography, including (**A**) photoresist coating, (**B**) UV exposure, (**C**) developing, (**D**) etching, and (**E**) stripping.

Figure 3 illustrates the primary stages a wafer undergoes before becoming an electrode (illustrated electrode is 38 × 26 × 1 mm^3^ with critical dimension of 30 microns). First, the gold-coated glass slide is cleaned to remove any contaminants that may be stuck to its surface. Then, the wafer is coated with positive photoresist using a spin coater, as shown in Figure 3(A). After being soft baked to evaporate the coating solvent, the glass slide is arranged with the photomask in a UV light box, as depicted in Figure 3(B). During the UV exposure stage, the regions of the exposed photoresist become soluble in the developer. Figure 3(C) shows the status of the glass after the developing step. At this stage, an examination of the glass slide under a microscope is strongly recommended to ensure that the overall structure was adequately created and to confirm that the exposed photoresist material washed away. Next, the glass slide is placed inside a convection oven for 45 minutes at 90 °C, in a stage called the “hard bake.” The goal of the hard bake is to stabilize and harden the developed photoresist. Next, the uncovered gold regions coated with photoresist are etched using a gold etching solution, until the glass slide appears, as shown in Figure 3(D). To make the gold electrode transparent, the seed layer is washed away. Finally, any remaining photoresist is removed, resulting in the final gold-coated glass electrode shown in Figure 3(E).

## 7. Ongoing Research

Currently, we are working on a new LOC design for cell manipulation and characterization. Specifically, this device will be used to conduct DEP experiments as a sample preparation prior to the stage of infectious diseases diagnosis.

The proposed design addresses the previously mentioned challenges with existing devices. We particularly wanted a design that met the following criteria: cost effectiveness, simple fabrication, leakage-free flow, reusability, the ability to change any component at any time, easy assembly, and compatibility with different gasket heights.

**Figure 4.**
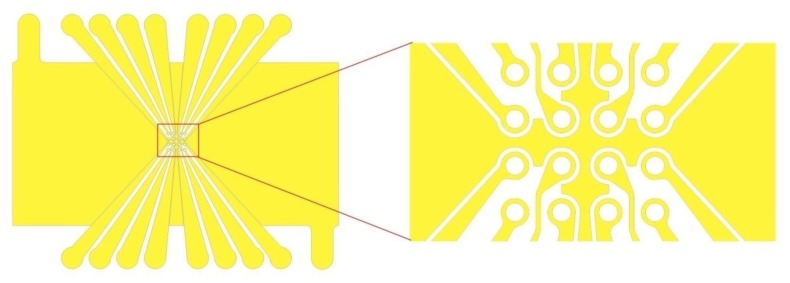
A schematic illustration of the proposed 4×4 microarray dot electrode geometry used in our LOC device.

In our proposed design, we chose to use the planar multiple microarray dot electrode (Figure 4) because of the various advantages discussed earlier. This electrode design is an improvement for the design proposed by Fatoyinbo *et al.* [8] described in Section 5. We have added ground plane in between the adjacent dots to avoid the overlapping between the electric fields generated by adjacent dots.

**Figure 5.**
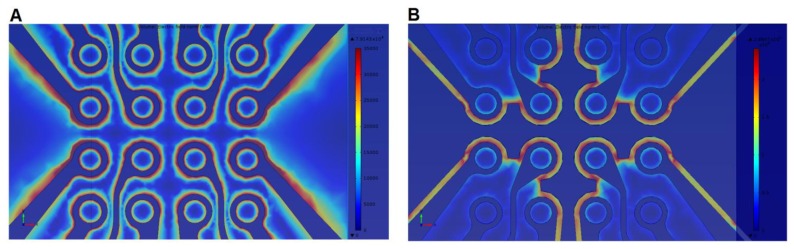
Simulation of electric field distribution over the dot electrode: (**A**) without ground plane between dot apertures; (**B**) with ground plane between dot apertures. Reproduced with permission from [74].

This electrode design had already been simulated in a previous work [74]. Figure 5 shows the simulation of the electric field strength over the dot electrode with and without ground plane between adjacent dot apertures. The electric field strength at the dot edge of the electrode without ground plane between adjacent dots was found 7.9 × 10^4^ V/m, whilst it increases to 2.9 × 10^5^ V/m when adding a ground plane between the adjacent dots. Results confirmed the capability of our proposed electrode design to produce higher electric field which leads to stronger DEP force.

**Figure 6.**
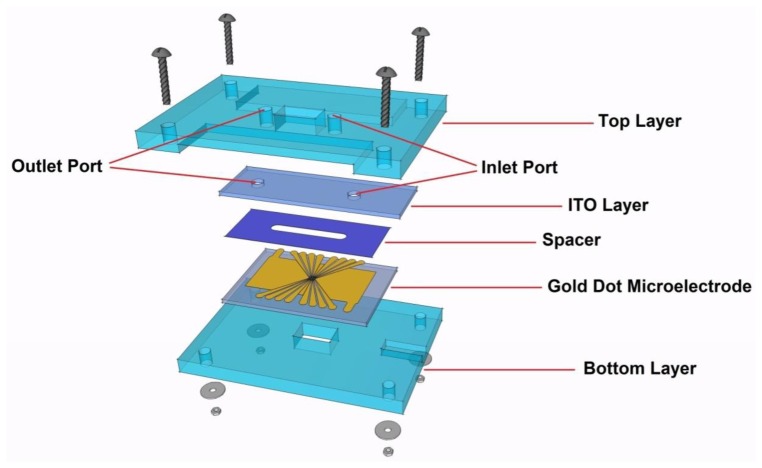
A schematic diagram of the proposed LOC device.

Our proposed LOC design consists of five layers, as shown in Figure 6. Detailed specifications on the dimensions and materials for each layer are described in Table 2. The ITO layer plays the role of a ground electrode; a unique advantage of ITO electrodes is their transparency, which facilitates DEP assay monitoring.

A spacer, where the DEP effect will take place, is another component of the device. A 3-mm channel should be cut at the middle of the spacer to create room for the fluid to flow. It is of great importance to fix the distance between the positive and ground electrodes throughout the microchannel, creating a symmetrical electric field distribution and avoiding any possible fluid leakage.

As shown in Figure 6, rectangular holes (10 mm × 6 mm) are cut through the top and bottom layers to allow better light transmission for observing and recoding the DEP effect under the microscope. The gold and ITO electrodes are connected to the function generator via flexible wires and silver-loaded epoxy. There are engraved areas in the top and bottom layers to provide a space for these connections, as illustrated in Figure 6.

## 8. Conclusions

Though DEP fields have been studied extensively, there is room for improvement. The significant quantity of research conducted on DEP in the last three decades has produced a wide range of microelectrode configurations. In this paper, we described a microarray dot electrode that could be a promising electrode geometry for cellular characterization and manipulation via DEP. The dot microelectrode has the potential to acquire the DEP spectra of biological cells in real-time using simple digital image processing techniques. The advantages offered by this type of microelectrode have been discussed above. The protocol for fabricating planar microelectrodes using photolithography has also been documented to demonstrate that it is a fast and cost-effective process. We also reviewed different state-of-the-art LOC devices that have been proposed in the literature for DEP applications. In addition, we presented our LOC device, which has been recently designed to address the challenges facing other devices. The proposed LOC device can be used to study the changes in a cell's electrical characteristics as they occur, enhancing our understanding of the role electrophysiology plays in drug actions. This study represents a step toward validating DEP in practical settings by proposing LOC designs that combine cost efficiency and ease of fabrication with rapid throughput. In the coming years, DEP technology will become a vital technique for many real and POC applications, freed from research laboratories.

## Figures and Tables

**Table 1.** The procedure for fabricating a gold microelectrode using a positive photoresist with the photolithography process.

**Step**	**Actions**
Step 1: *Surface Preparation*	Soak the gold-coated glass slides first in acetone, then in methanol and then in DI H_2_O with 5 minutes of ultrasonic agitation at each step. Then, dry the slides on a hotplate at 120 °C for 10 minutes.

Step 2: *Photoresist Coating*	Coat the slides with a positive photoresist using a vacuum spin coater. Set the spin coater parameters to 3,000 rpm and 30 seconds.

Step 3: *Soft Bake*	Lay the slides on a hotplate for 60 seconds at 100 °C.

Step 4: *UV Exposure*	Expose the slides to UV light through the photomask for 40 seconds.

Step 5: *Developing*	Immerse the exposed slides into the developer solution for a few seconds only, and then rinse the slides with DI H_2_O to avoid an overreaction. A longer developing time has an adverse effect on the unexposed gold layer.

Step 6: *Hard Bake*	Place the slides in a convection oven for 45 minutes at 90 °C.

Step 7: *Etching*	Dip the slides into gold etchant, removing them once the exposed gold has been washed away. Rinse the slides with DI H_2_O.

Step 8: *Seed Layer Removal*	Place the etched slides in boiled 18% HCl until the seed layer has bubbled away. Rinse the slides with DI H_2_O.

Step 9: *Stripping*	Remove any leftover photoresist using a positive photoresist stripper solution for 5–10 seconds. Rinse the slides with DI H_2_O and dry them.

Step 10: *Disposal*	Dispose of all solutions safely.

**Table 2.** Design specifications of the different layers of the proposed LOC device.

**Component**	**Material**	**Dimension (mm)**		

		*Long*	*Width*	*Thickness*
Top Layer	PMMA	60	40	4
ITO Layer	ITO	45	15	1
Spacer	Polyresin	38	15	0.1
Electrode	Gold	38	26	1
Bottom Layer	PMMA	60	40	4

## References

[1] Ritzi-Lehnert M. (2012). Development of chip-compatible sample preparation for diagnosis of infectious diseases. Expert Rev. Mol. Diagn..

[2] Haeberle S., Zengerle R. (2007). Microfluidic platforms for lab-on-a-chip applications. Lab Chip.

[3] Sin M., Gao J., Liao J., Wong P. (2011). System integration—A major step toward lab on a chip. J. Biol. Eng..

[4] Masuda S., Washizu M., Nanba T. (1989). Novel method of cell fusion in field constriction area in fluid integration circuit. IEEE Trans. Indust. Appl..

[5] Gascoyne P.R.C., Xiao-Bo W., Ying H., Becker F.F. (1997). Dielectrophoretic separation of cancer cells from blood. IEEE Trans. Indust. Appl..

[6] Gasperis G., Yang J., Becker F., Gascoyne P.C., Wang X.-B. (1999). Microfluidic Cell Separation by 2-dimensional Dielectrophoresis. Biomed. Microdevices.

[7] Li H., Bashir R. (2002). Dielectrophoretic separation and manipulation of live and heat-treated cells of Listeria on microfabricated devices with interdigitated electrodes. Sens. Actuators B: Chem..

[8] Fatoyinbo H.O., Kadri N.A., Gould D.H., Hoettges K.F., Labeed F.H. (2011). Real time cell electrophysiology using a multi channel dielectrophoretic dot microelectrode array. Electrophoresis.

[9] Bousse L., Cohen C., Nikiforov T., Chow A., Kopf-Sill A.R., Dubrow R., Parce J.W. (2000). Electrokinetically controlled microfluidic analysis systems. Annu. Rev. Biophys. Biomol. Struc..

[10] Pohl H.A. (1951). The motion and precipitation of suspensoids in divergent electric fields. J. Appl. Phys..

[11] Pohl H.A., Hawk I. (1966). Separation of living and dead cells by dielectrophoresis. Science.

[12] Gagnon Z.R. (2011). Cellular dielectrophoresis: applications to the characterization, manipulation, separation and patterning of cells. Electrophoresis.

[13] Voldman J. (2006). Electrical forces for microscale cell manipulation. Annu. Rev. Biomed. Eng..

[14] Kadri N.A. (2011). Development of Near Real-Time Assessment System for Cancer Cells. Ph.D. Thesis.

[15] Archer S., Morgan H., Rixon F.J. (1999). Electrorotation studies of baby hamster kidney fibroblasts infected with herpes simplex virus type 1. Biophys. J..

[16] Green N., Morgan H. (1999). Dielectrophoretic separation of nano-particles. J. Phys. D: Appl. Phys..

[17] Hughes M.P., Morgan H. (1998). Dielectrophoretic trapping of single sub-micrometre scale bioparticles. J. Phys. D: Appl. Phys..

[18] Morgan H., Hughes M.P., Green N.G. (1999). Separation of submicron bioparticles by dielectrophoresis. Biophys. J..

[19] Masuda T., Maruyama H., Honda A., Araf F. Virus Enrichment for Single Virus Manipulation by Using 3D Insulator Based Dielectrophoresis.

[20] Nakano A., Ros A. (2013). Protein dielectrophoresis: Advances, challenges and applications. Electrophoresis.

[21] Clarke R.W., White S.S., Zhou D., Ying L., Klenerman D. (2005). Trapping of Proteins under Physiological Conditions in a Nanopipette. Angew. Chemie.

[22] Zheng L., Brody J.P., Burke P.J. (2004). Electronic manipulation of DNA, proteins, and nanoparticles for potential circuit assembly. Biosens. Bioelectron..

[23] Gascoyne P., Mahidol C., Ruchirawat M., Satayavivad J., Watcharasit P., Becker F.F. (2002). Microsample preparation by dielectrophoresis: isolation of malaria. Lab Chip.

[24] Markx G.H., Huang Y., Zhou X.-F., Pethig R. (1994). Dielectrophoretic characterization and separation of micro-organisms. Microbiology.

[25] Suehiro J., Noutomi D., Shutou M., Hara M. (2003). Selective detection of specific bacteria using dielectrophoretic impedance measurement method combined with an antigen–antibody reaction. J. Electrostat..

[26] Hamada R., Takayama H., Shonishi Y., Mao L., Nakano M., Suehiro J. (2013). A rapid bacteria detection technique utilizing impedance measurement combined with positive and negative dielectrophoresis. Sens. Actuators B: Chem..

[27] Henning A., Bier F.F., Hölzel R. (2010). Dielectrophoresis of DNA: Quantification by impedance measurements. Biomicrofluidics.

[28] Gallo-Villanueva R.C., Rodríguez-López C.E., Díaz-de-la-Garza R.I., Reyes-Betanzo C., Lapizco-Encinas B.H. (2009). DNA manipulation by means of insulator-based dielectrophoresis employing direct current electric fields. Electrophoresis.

[29] Martinez-Duarte R., Camacho-Alanis F., Renaud P., Ros A. (2013). Dielectrophoresis of lambda-DNA using 3D carbon electrodes. Electrophoresis.

[30] Fatoyinbo H.O., Hughes M.P., Martin S.P., Pashby P., Labeed F.H. (2006). Dielectrophoretic separation of Bacillus subtilis spores from environmental diesel particles. J. Environ. Monit..

[31] Koklu M., Park S., Pillai S.D., Beskok A. (2010). Negative dielectrophoretic capture of bacterial spores in food matrices. Biomicrofluidics.

[32] Hübner Y., Hoettges K.F., Hughes M.P. (2003). Water quality test based on dielectrophoretic measurements of fresh water algae Selenastrum capricornutum. J. Environ. Monit.

[33] Song Y., Yang J., Shi X., Jiang H., Wu Y., Peng R., Wang Q., Gong N., Pan X., Sun Y. (2012). DC dielectrophoresis separation of marine algae and particles in a microfluidic chip. Sci. Chin. Chem..

[34] Dalton C., Goater A., Drysdale J., Pethig R. (2001). Parasite viability by electrorotation. Colloid Surface Physicochem. Eng. Aspect.

[35] Menachery A., Kremer C., Wong P.E., Carlsson A., Neale S.L., Barrett M.P., Cooper J.M. (2012). Counterflow Dielectrophoresis for Trypanosome Enrichment and Detection in Blood.

[36] Pethig R., Talary M.S. (2007). Dielectrophoretic detection of membrane morphology changes in Jurkat T-cells undergoing etoposide-induced apoptosis. IET Nanobiotechnol..

[37] Nikolic-Jaric M., Cabel T., Salimi E., Bhide A., Braasch K., Butler M., Bridges G.E., Thomson D.J. (2013). Differential electronic detector to monitor apoptosis using dielectrophoresis-induced translation of flowing cells (dielectrophoresis cytometry). Biomicrofluidics.

[38] Mernier G., Piacentini N., Tornay R., Buffi N., Renaud P. (2009). Label-free Sorting and Counting of Yeast Cells for Viability Studies. Procedia Chem..

[39] Mernier G., Piacentini N., Tornay R., Buffi N., Renaud P. (2011). Cell viability assessment by flow cytometry using yeast as cell model. Sensor. Actuators B: Chem..

[40] Young C.-W., Hsieh J.-L., Ay C. (2012). Development of an Integrated Chip for Automatic Tracking and Positioning Manipulation for Single Cell Lysis. Sensors.

[41] Chiou C.-H., Pan J.-C., Chien L.-J., Lin Y.-Y., Lin J.-L. (2013). Characterization of Microparticle Separation Utilizing Electrokinesis within an Electrodeless Dielectrophoresis Chip. Sensors.

[42] Yunus N.A.M., Nili H., Green N.G. (2013). Continuous separation of colloidal particles using dielectrophoresis. Electrophoresis.

[43] Washizu M., Suzuki S., Kurosawa O., Nishizaka T., Shinohara T. (1994). Molecular dielectrophoresis of biopolymers. IEEE Trans. Indust. Appl..

[44] Jaber F.T., Labeed F.H., Hughes M.P. (2009). Action potential recording from dielectrophoretically positioned neurons inside micro-wells of a planar microelectrode array. J. Neurosci. Meth..

[45] Imasato H., Yamakawa T., Eguchi M. (2012). Separation of Leukemia Cells from Blood by Employing Dielectrophoresis. Intell. Autom. Soft Comput..

[46] Patel S., Showers D., Vedantam P., Tzeng T.-R., Qian S., Xuan X. (2012). Microfluidic separation of live and dead yeast cells using reservoir-based dielectrophoresis. Biomicrofluidics.

[47] Piacentini N., Mernier G., Tornay R., Renaud P. (2011). Separation of platelets from other blood cells in continuous-flow by dielectrophoresis field-flow-fractionation. Biomicrofluidics.

[48] Chuang C.-H., Huang Y.-W., Wu Y.-T. (2011). System-level biochip for impedance sensing and programmable manipulation of bladder cancer cells. Sensors.

[49] Rosales-Cruzaley E., Cota-Elizondo P., Sánchez D., Lapizco-Encinas B.H. (2012). Sperm cells manipulation employing dielectrophoresis. Bioprocess. Biosyst. Eng..

[50] Pethig R., Markx G.H. (1997). Applications of dielectrophoresis in biotechnology. Trends Biotechnol..

[51] Cao J., Cheng P., Hong F. (2008). A numerical analysis of forces imposed on particles in conventional dielectrophoresis in microchannels with interdigitated electrodes. J. Electrostat..

[52] Lapizco-Encinas B.H., Davalos R.V., Simmons B.A., Cummings E.B., Fintschenko Y. (2005). An insulator-based (electrodeless) dielectrophoretic concentrator for microbes in water. J. Microbiol. Meth..

[53] Chou C.-F., Tegenfeldt J.O., Bakajin O., Chan S.S., Cox E.C., Darnton N., Duke T., Austin R.H. (2002). Electrodeless dielectrophoresis of single-and double-stranded DNA. Biophy. J..

[54] Khoshmanesh K., Nahavandi S., Baratchi S., Mitchell A., Kalantar-zadeh K. (2011). Dielectrophoretic platforms for bio-microfluidic systems. Biosen. Bioelectron..

[55] Castellanos A., Ramos A., Gonzalez A., Green N.G., Morgan H. (2003). Electrohydrodynamics and dielectrophoresis in microsystems: scaling laws. J. Phys. D: Appl. Phys..

[56] Liu L., Ye X., Wu K., Han R., Zhou Z., Cui T. (2009). Humidity sensitivity of multi-walled carbon nanotube networks deposited by dielectrophoresis. Sensors.

[57] Becker F.F., Wang X.B., Huang Y., Pethig R., Vykoukal J., Gascoyne P. (1995). Separation of human breast cancer cells from blood by differential dielectric affinity. Proc. Nat. Acad. Sci. USA.

[58] Wang X.B., Huang Y., Wang X., Becker F.F., Gascoyne P. (1997). Dielectrophoretic manipulation of cells with spiral electrodes. Biophys. J..

[59] Khoshmanesh K., Zhang C., Tovar-Lopez F.J., Nahavandi S., Baratchi S., Kalantar-zadeh K., Mitchell A. (2009). Dielectrophoretic manipulation and separation of microparticles using curved microelectrodes. Electrophoresis.

[60] Pommer M.S., Zhang Y., Keerthi N., Chen D., Thomson J.A., Meinhart C.D., Soh H.T. (2008). Dielectrophoretic separation of platelets from diluted whole blood in microfluidic channels. Electrophoresis.

[61] Jang L.S., Huang P.H., Lan K.C. (2009). Single-cell trapping utilizing negative dielectrophoretic quadrupole and microwell electrodes. Biosens. Bioelectron..

[62] Suehiro J., Pethig R. (1998). The dielectrophoretic movement and positioning of a biological cell using a three-dimensional grid electrode system. J. Phys. D: Appl. Phys..

[63] Thomas R.S., Morgan H., Green N.G. (2009). Negative DEP traps for single cell immobilisation. Lab Chip.

[64] Hoettges K.F., Hübner Y., Broche L.M., Ogin S.L., Kass G.E.N., Hughes M.P. (2008). Dielectrophoresis-activated multiwell plate for label-free high-throughput drug assessment. Anal. Chem..

[65] Iliescu C., Yu L., Tay F.E.H., Chen B. (2008). Bidirectional field-flow particle separation method in a dielectrophoretic chip with 3D electrodes. Sensor. Actuators B: Chem..

[66] Wang L., Lu J., Marchenko S.A., Monuki E.S., Flanagan L.A., Lee A.P. (2009). Dual frequency dielectrophoresis with interdigitated sidewall electrodes for microfluidic flow-through separation of beads and cells. Electrophoresis.

[67] Dürr M., Kentsch J., Müller T., Schnelle T., Stelzle M. (2003). Microdevices for manipulation and accumulation of micro-and nanoparticles by dielectrophoresis. Electrophoresis.

[68] Pohl H.A., Plymale C.E. (1960). Continuous separations of suspensions by nonuniform electric fields in liquid dielectrics. J. Electrochem. Soc..

[69] Chung C.-C., Cheng I.F., Chen H.-M., Kan H.-C., Yang W.-H., Chang H.-C. (2012). Screening of antibiotic susceptibility to β-Lactam-Induced Elongation of gram-negative bacteria based on dielectrophoresis. Anal. Chem..

[70] Moghimi N., Decker D.R., Tatic-Lucic S. (2012). Modeling and measurement of dielectrophoretic force and 2-D trajectories of microspheres in quadrupole electrode configuration. IEEE Sens. J..

[71] Guan W., Joseph S., Park J.H., Krstić P.S., Reed M.A. (2011). Paul trapping of charged particles in aqueous solution. Proc. Nat. Acad. Sci. USA.

[72] Voldman J., Toner M., Gray M., Schmidt M. (2003). Design and analysis of extruded quadrupolar dielectrophoretic traps. J. Electrostat..

[73] Taff B.M., Voldman J. (2005). A scalable addressable positive-dielectrophoretic cell-sorting array. Anal. Chem..

[74] Yafouz B., Kadri N.A., Ibrahim F. (2012). The Design and Simulation of a Planar Microarray Dot Electrode for a Dielectrophoretic Lab-on-Chip Device. Int. J. Electrochem. Sci.

[75] Fatoyinbo H.O., Hoettges K.F., Hughes M.P. (2008). Rapid-on-chip determination of dielectric properties of biological cells using imaging techniques in a dielectrophoresis dot microsystem. Electrophoresis.

[76] Stevens K.A., Jaykus L.-A. (2004). Bacterial separation and concentration from complex sample matrices: a review. Critical Rev. Microbiol..

[77] Park S., Koklu M., BeskoK A. (2009). Particle trapping in high-conductivity media with electrothermally enhanced negative dielectrophoresis. Anal. Chem..

[78] Fiorini G.S., Chiu D.T. (2005). Disposable microfluidic devices: Fabrication, function, and application. BioTechniques.

